# Editorial: Brain-connectivity-based computer interfaces

**DOI:** 10.3389/fnhum.2023.1281446

**Published:** 2023-09-05

**Authors:** Ilaria Boscolo Galazzo, Luca Tonin, Aleksandar Miladinović, Silvia Francesca Storti

**Affiliations:** ^1^Department of Engineering for Innovation Medicine, University of Verona, Verona, Italy; ^2^Department of Information Engineering, University of Padua, Padua, Italy; ^3^Padova Neuroscience Center, University of Padua, Padua, Italy; ^4^Institute for Maternal and Child Health - IRCCS Burlo Garofolo, Trieste, Italy

**Keywords:** BCI, EEG, brain connectivity, dynamic functional connectivity, AI

Over the past few decades, brain-computer interfaces (BCIs) have undergone significant expansion, driven by innovative methodologies and technological advancements. Among the emerging methodologies that promote new BCI models, brain connectivity stands out. These systems hold the potential to completely reshape the interactions with technology and, importantly, to redefine our approach to addressing neurological conditions. BCIs are primarily distinguished by their invasive or non-invasive recording methods, such as electroencephalography (EEG), magnetoencephalography MEG, functional near-infrared spectroscopy (fNIRS), functional magnetic resonance imaging (fMRI) and stereo EEG, as well as their targeted applications.

In the medical field, BCIs hold a crucial role in *communication* and *neurorehabilitation*, aiming to restore the ability to communicate or recover abilities that have been lost. This mission is particularly important in the rehabilitation context, where interfaces can be used to facilitate central functional recovery, especially for individuals recovering from stroke (López-Larraz et al., 2018). The works of Liao et al. and de Seta et al. have taken steps in this direction. Liao et al. have combined motor-imagery BCI (MI-BCI) with physiotherapy, creating a synergy between technology and recovery methodologies. The central focus was to probe whether the impact of MI-BCI varies with patient severity and if it provides universal recovery benefits. To unveil the effectiveness of this innovative approach, the researchers recruited a cohort of hospitalized ischemic stroke patients who exhibited motor deficits. They used standard tests before and after the rehabilitation along with non-contrast CT scans to assess the effects of high-density signs on the prognosis of stroke. The dynamic changes in neural activity after stroke were mapped out using brain topographic maps. The findings highlighted the superior performance of MI-BCI compared to conventional rehabilitation methods.

BCIs should accommodate individual needs, using residual signals that can originate not only from the brain but also from the muscles. These innovative technologies, called hybrid BCIs (h-BCIs), bridge the gap between cerebral and muscular signals, as described by the work of de Seta et al.. The authors have established a novel approach to enhance post-stroke motor rehabilitation by focusing on cortico-muscular coupling (CMC) within a h-BCI. Through a pseudo-online analysis involving both healthy and stroke subjects, the study optimizes CMC computation and its translation into movement detection. This refinement, including adjusting CMC calculations every 125 ms and collecting two predictions before making a final decision, greatly enhances both accuracy and speed, no matter the type of movement. Importantly, the attempts and actual movements of stroke patients are both classified using CMC-based detection, achieving high accuracy and rapid classification. These findings lay the groundwork for a novel non-invasive h-BCI design, leveraging combined EEG and EMG connectivity patterns during upper limb movement attempts.

While most of the ongoing research is focusing on upper body movements, as demonstrated by the previously discussed studies, it is important to mention that BCI techniques can also target lower extremities, although decoding lower limb movements from EEG brain signals is more challenging. As such, EEG for lower extremity prosthetic control is currently rare (Lennon et al., 2020). The study by Dillen et al. aims at making a step forward in this respect and proposes different data-driven approaches to investigate the feasibility of lower limb BCI in participants with an amputation and able-bodied participants performing lower limb movements. Different machine learning (ML) methods are tested, such as random forest, logistic regression, and linear discriminant analysis, each combined with a series of features based on power spectral density, common spatial patterns and xDAWN algorithm. The authors demonstrate that EEG can be reliably used as a control signal for lower limb prostheses in BCIs, with random forest methods appearing as the best classifier choice in most of the cases and common spatial patterns as the most suitable feature extraction method. Further integrating EEG with other signals (as EMG) could be of high interest for designing accurate h-BCI systems targeting lower limb prosthetic device control.

In this panorama, the recent introduction of the *brain connectivity* concept, borrowed from the fields of neurophysiology and neuroimaging, has led to a revolution and substantial enhancement in brain-device communication, albeit still accompanied by several usability challenges, particularly in real-time scenarios (Behrens and Sporns, 2012; Finn et al., 2015; Siviero et al., 2023). The quantification of brain connectivity assumes different forms, allowing for the assessment of relationships among distinct brain regions, as well as between the brain and other parts of the body, muscles included. This can be exemplified thought functional and effective connectivity analyses. The former involves assessing the temporal correlation of activity between different brain areas. The latter, demonstrated by techniques like Granger causality or dynamic causal modeling, explores the directional influence that one brain region exerts over another. Their use has facilitated the selection of a wider set of features, which not only describe the functionality of brain regions but also capture their intricate interactions. These features have proven pivotal in decoding cerebral functions and subsequently translating them into active commands through the BCI interface (Hamedi et al., 2016; Brusini et al., 2021). For example, the analysis of brain connections empowers BCIs to discern specific activity patterns linked to finer or more complex movements. This holds particular significance for real-time command classification. Another pivotal aspect concerns the integration of *dynamic connectivity*, both in a general sense and when applied to the connectivity metrics themselves. This encompasses an exploration of how connections between distinct brain areas evolve over time. Consequently, BCIs have gained the ability to adapt to shifts in brain function, amplifying their effectiveness in comprehending user commands as time progresses.

Connectivity-based features are also relevant in neurofeedback training applications. These allow self-regulation of one's own brain activity by providing feedback and reward signals, representing one of the earliest and simplest applications of BCI systems (Birbaumer et al., 2009). Neurofeedback has been increasingly suggested as a potential complementary therapy in different disorders, including epilepsy, attention deficit disorder, depression, and schizophrenia (Marzbani et al., 2016). However, as outlined by Trambaiolli et al., not all the individuals will benefit from neurofeedback training, and it is thus essential to identify potential predictors of performance and outcomes. Through their study, authors combine functional connectivity (FC) features from simultaneous EEG and fNIRS recordings to predict participants' performance in an fNIRS-based affective neurofeedback task on healthy participants. Their results provide evidence of the feasibility of using multimodal FC predictors for neurofeedback performance, revealing good prediction accuracy and demonstrating good agreement between resting-state FC connectomes derived from different functional modalities.

Currently, brain connectivity and network analysis on EEG data have experienced rapid advancement, from simple temporal correlations among brain regions to complex models encompassing dynamic and causal effects. However, as spatial and temporal precision improve, data dimensionality increases, intensifying the challenges of analysis. Furthermore, the modeling of brain networks is complex due to non-linear neuronal interactions, sometimes eluding traditional linear models. Although advanced ML and deep learning (DL) methods appear promising for such purpose and for dealing with high dimensionality, the intrinsically noisy, sparse, and non-stationary nature of EEG data imposes several limitations. Moreover, many ML/DL approaches often lack transparency and frequently remain as “black boxes”, generating results without explanations. Therefore, the current development and use of *explainable artificial intelligence* (XAI) is highly relevant, allowing users to better understand how models make decisions and increasing confidence in the system. However, the efficacy of AI-based approaches comes with a caveat. They require a large amount of data for training, and this may represent a substantial challenge within the BCI context, especially in clinical and rehabilitative applications. In these scenarios, limited samples sizes and the need to adapt the system to each user are peculiar. To overcome such limitations, several potential solutions could be considered. For instance, leveraging publicly available data could partially mitigate the data scarcity issue. Similarly, using approaches such as *transfer learning* could be useful in addressing this problem allowing the transfer of knowledge from domains with a large amount of data to those with more limited datasets (Lotte et al., 2018).

To conclude, brain connectivity analysis, dimensionality reduction, modeling of non-linear interactions, integration of different data sources for h-BCI, individually-tailored systems and the pursuit of transparent AI represent essential topics to further explore that all converge in current BCI research, with a greater challenge lying in achieving real-time implementations.

## Author contributions

IBG: Writing—original draft, Writing—review and editing. LT: Writing—review and editing. AM: Writing—review and editing. SS: Writing—original draft, Writing—review and editing, Conceptualization.
